# Japanese Traditional *Miso* and *Koji* Making

**DOI:** 10.3390/jof7070579

**Published:** 2021-07-20

**Authors:** Ken-Ichi Kusumoto, Youhei Yamagata, Rina Tazawa, Manabu Kitagawa, Taeko Kato, Kenji Isobe, Yutaka Kashiwagi

**Affiliations:** 1 Food Research Institute, National Agriculture and Food Research Organization, Tsukuba 305-8642, Japan; kusumoto@affrc.go.jp; 2 Division of Applied Biological Chemistry, Institute of Agriculture, Tokyo University of Agriculture and Technology, Fuchu, Tokyo 183-8509, Japan; y-yama@cc.tuat.ac.jp; 3 Marukome Co., Ltd., Nagano 380-0943, Japan; rina_tazawa@marukome.co.jp (R.T.); manabu_kitagawa@marukome.co.jp (M.K.); 4 Central Miso Research Institute, Chuo-ku, Tokyo 104-0033, Japan; 1089-kato@miso.or.jp; 5 Japan Federation of Miso Manufacturers Cooperatives, Chuo-ku, Tokyo 104-0033, Japan; 6 Department of Fermentation Science, Tokyo University of Agriculture, Setagaya-ku, Tokyo 156-8502, Japan; y3kashiw@nodai.ac.jp

**Keywords:** *Aspergillus oryzae*, *koji*, *miso*, soybean, enzymes, peptidase, fermentation

## Abstract

*Miso* is a traditional Japanese seasoning paste produced by fermenting soybeans using the power of *koji* mold. A recent Japanese cohort study has shown that increased consumption of fermented soybean products is associated with a reduced risk of death in both men and women. In this review, we briefly explain what *miso* means in the Japanese culture and food industry, varieties of *miso* available today, and steps involved in *miso* making. Then, we review early and latest scientific researches in *koji* mold species, their safety, and beneficial enzymes they produce during fermentation and maturation processes, which play a major part in determining the quality and sensory profile of *miso*.

## 1. Introduction

The earliest form of *miso* called *kokusho* (soybeans and grains fermented with salt) is said to have originated from ancient China or perhaps in Japan thousands of years ago [1]. The use of *miso* spread among Japanese people during the Edo Period (1603–1868), and *miso* constitutes one of the hallmarks of the country’s salted and fermented soybean seasoning along with soy sauce. *Miso* was integrated into local food cultures across Japan and evolved into various types, reflecting regional differences in climate and ingredient availability [2,3]. This is the reason *miso* comes in so many varieties with different colors and flavors. Today, rice *miso* is the most manufactured variety, while barley, soybean, and mixed *miso* are also available.

This review describes the manufacturing process of *miso*, including the critical step of *koji* making, microbes used for fermentation, and enzymes found in *koji*. Some of the latest topics on regular consumption of *miso* and its health benefits are also discussed.

## 2. *Miso* Varieties and Its Culinary Scene

*Miso* is regulated by the Food Labeling Standards as specified by the Food Labeling Act [4]. *Miso* is defined as a semisolid paste primarily made from soybeans, which are combined, fermented, and matured with soybeans and/or grains cultured with *koji* mold and salt. According to the ingredients used, *miso* is classified into four types: rice, barley, soybean, and mixed *miso*. Rice *miso* is made from rice, soybeans, and salt. Rice is first fermented with *koji* mold to produce *koji*, which is then used for the fermentation and maturation of soybeans. Barley *miso* is produced in much the same way, except that barley or naked barley is used instead of rice. Soybean *miso* is made from soybeans and salt using soybean *koji* for fermentation and maturation. Mixed *miso* can be any combination of rice, barley, and/or soybean *miso* or any *miso* produced using a mixture of rice, barley, and/or soybean *koji*. *Miso* can also be classified by taste and color. The proportion of *koji* (the rice-to-soybean ratio for rice *miso* and the barley-to-soybean ratio for barley *miso*) determines the sweetness. Higher proportions of rice or barley *koji* create a sweeter taste, while lower proportions of *koji* produce saltiness. There are red, yellow, and white *miso* according to the color of the finished product.

The most typical use of *miso* in Japanese cuisine is *miso* soup. Seasonal ingredients (vegetables, seaweed, and seafood) are cooked in *dashi* soup stock made from dried bonito, dried kelp, or other flavoring ingredients, and a spoonful of *miso* paste is dissolved in the soup.

In 2013, *washoku* was added to UNESCO’s Intangible Cultural Heritage List as traditional dietary cultures of the Japanese. Extending in a north-to-southwardly direction, Japan has a rich and diverse natural environment with four distinct seasons. *Washoku* is a cooking and serving practice that was born and nurtured in this unique environment and essentially represents Japanese people’s spirit of respect for nature [5]. The taste of *miso* is formed by complex interactions of sweetness, saltiness, *umami*, acidity, bitterness, and astringency. It can mask meaty and fishy odors, while adding *umami* and depth to a variety of dishes. *Miso* is one of the most fundamental fermented seasoning at the heart of *washoku* and essential to the everyday diet of Japanese people. It is also a part of the “One Soup Three Dishes” principle of *washoku* serving. Japanese people feel a sense of nostalgia and hometown familiarity along with comfort and warmth when they drink *miso* soup.

## 3. Process of *Miso* Making

Rice *miso* is the most common variety produced throughout Japan and accounts for as high as about 80% of the total production [6]. On the other hand, barley *miso* is manufactured mainly in the southern parts of Japan—Kyushu, Shikoku, and Chugoku regions, and soybean *miso* is mainly preferred in Chubu, the middle part of Japan.

**Figure 1:**
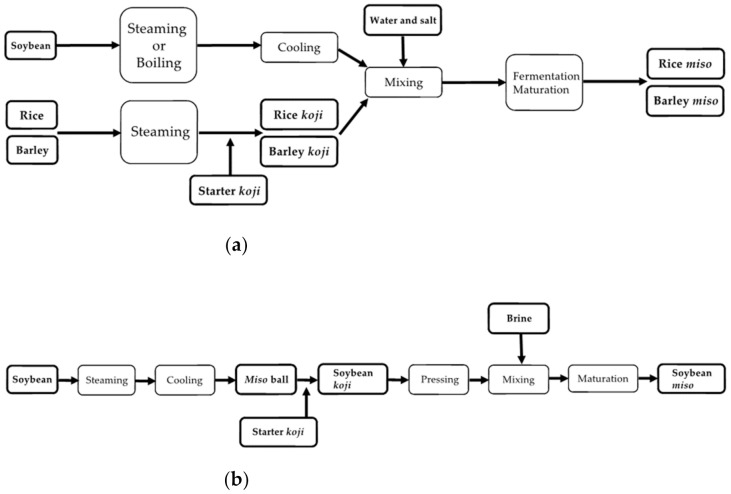
Overview of the *miso* making process for (**a**) rice and barley *miso* and (**b**) soybean *miso*.

Since old times, *koji* making has been considered the most critical step, followed by soybean processing and mixing, among many steps of *miso* making [7]. Figure 1 summarizes these steps in *miso* making.

### 3.1. Rice Miso

The following explains the process of rice *miso* making [8,9]. Soybeans are washed and soaked in water typically for 15 to 17 h. In the next step, they are either steamed or boiled usually under pressure. After steaming or boiling, soybeans are then cooled down.

Rice *koji* making starts from the selection of rice grains and soaking followed by steaming for complete conversion of raw starch (β-starch) to gelatinized starch (α-starch). Near-saturated steam is used for 40 to 60 min. Steamed rice is cooled and then inoculated with *koji* mold. Although this differs depending on the culture room conditions, the first *teire* or mixing (discussed later) is usually done around 18 h after inoculation and the second *teire* usually around 26 h. It takes a total of 42 to 48 h to produce rice *koji*. After inoculation, the temperature is maintained at 28–32 °C during loading into the culture room (or *hikikomi*; see below) and at 40 °C or below during *koji* fermentation.

Mixing *miso* ingredients is called *shikomi*. Rice *koji*, steamed or boiled soybeans, water, salt, yeast, and lactic acid bacteria are mixed thoroughly and transferred into a fermentation/maturation tank. Steamed or boiled soybeans are crushed before mixing, typically by processing through a chopper with a 5–6 mm mesh. They are then immediately mixed with rice *koji*, salt water, and other ingredients. Generally, salt-tolerant yeast *Zygosaccharomyces rouxii* and salt-tolerant lactic acid bacterium *Tetragenococcus halophilus* are used for *shikomi*.

Mixed ingredients are fermented and matured in a tank. Temperature control and the timing of inversion called *tenchi-gaeshi* are essential steps during fermentation and maturation. *Tenchi-gaeshi* is a process in which *miso* is transferred upside down into another tank. The purpose is to

(1) Ensure uniform fermentation and maturation across all parts of the tank;
(2) Provide aeration to facilitate the yeast’s aerobic fermentation and growth (yeast does not grow under anaerobic conditions, although it generates alcohol that affects the flavor);
(3) Release fermentation heat.

Fermentation and maturation are greatly affected by the enzymatic activities of *koji* and temperature of *miso*. For *miso* production based on both enzymatic and microbial activities, the temperature is maintained between 25 and 30 °C. The length of the maturation period is determined by what type of *miso* we want to produce and varies from 1 month to years.

This is how *miso* is made through fermentation. There is another type of *miso*, which is produced based on enzymatic degradation. There are two varieties of “enzymatically degraded” *miso*: white sweet types such as Kyoto white *miso* and red sweet types such as Edo sweet *miso*. These *miso* use higher percentages of *koji*, and they are sweeter and lower in salt (5 to 7%). The maturation takes place solely through *koji* enzyme activities and completes within a matter of several days at around 50 °C of controlled temperature (high-temperature digestion).

### 3.2. Barley Miso

The following explains the process of barley *miso* making [8]. Barley *miso* is produced much like rice *miso* with the following specific modifications.

Because barley absorbs water very quickly, the optimal soaking time is defined for each water temperature. During soaking, barley grains swell and form hard and tight clumps that must be loosened. They also form clumps during steaming and have to be broken up before loading into the *koji* facility to prevent uneven growth of the *koji* mold. The barley *koji* making process is similar to that of rice *koji* except that the higher protein content of barley makes it more susceptible to bacterial contamination, higher exothermic temperature, and clumping of *koji*.

There are mainly two types of barley *miso*: (1) light-colored *miso* with a higher proportion of *koji*, shorter maturation period, characteristic *koji* aroma, and salt content of 9 to 11%, and (2) red *miso* with a longer maturation period and higher salt content of 10 to 12%.

### 3.3. Soybean Miso

The following explains the process of soybean *miso* making [8]. Soybeans and salt are the only ingredients in soybean *miso*. Soybeans are washed and then soaked. The soaking process is the critical point that directly determines the product quality. The restricted water absorption technique is used to strictly control the amount of water and soaking time to produce soybeans with an ideal moisture content after steaming for the inoculation of *koji* mold. In general, the optimal weight of soaked and drained soybeans is 1.5 to 1.6 times the original weight. After soaking, soybeans are usually steamed under pressure.

The first step of *koji* making is to make *miso* balls. Steamed soybeans are cooled and then molded into balls using a specialized machine. These balls are inoculated with a *koji* starter and transferred to a *koji* facility. The size of a *miso* ball ranges from 15 to 40 mm in diameter and influences the quality of finished *miso*. Small *miso* balls tend to allow higher enzymatic activities, while larger *miso* balls have higher lactic acid contents, which give a fresher taste. *Aspergillus oryzae* or *Aspergillus sojae* is typically used as a starter.

The key point in soybean *koji* making is to maintain low temperatures to promote the growth of *koji* mold and lactic acid bacteria while suppressing *Bacillus* spp. The temperature is maintained at 27–28 °C in the initial phase. After germination of *koji* spores, the *koji* temperature is increased gradually up to 35–37 °C and generally maintained at 33–35 °C in the later phase.

For *shikomi*, soybean *koji* is pressed with a roller mill and mixed with saturated salt water and additional salt.

Soybean *miso* is matured for at least 6 to 12 months without heat treatment and around 4 to 6 months with heat treatment.

## 4. Filamentous Fungi, *Koji* Mold, in Japanese Fermented Soybean Paste

### 4.1. Brewer’s Koji Mold

#### 4.1.1. *Koji* Mold for *Sake*, *Miso* and Soy Sauce

*Koji* mold is a filamentous fungus used in the making of a variety of fermented foods such as *sake* (rice wine), soy sauce, *miso*, and *mirin*. It belongs to *Aspergillus oryzae*, also known as, yellow *koji* mold, which can be further classified into many varieties or strains depending on the application. Brewer’s starter *koji* is commercially available as *koji* spores and marketed by starter *koji* manufacturers. To manufacture a starter *koji*, a stock *koji* strain is inoculated to steamed brown rice or wheat bran and cultured for a period longer than typical *koji* mold culture to ensure good sporulation. Spores are then dried and harvested. Some *koji* starters contain cereals used as the growth medium. In others, spores are separated by sieving and sold as a uniform powder after the addition of a bulking agent such as starch and calcium carbonate.

*Koji* mold for *sake* making is characterized by a higher capability to produce amylase, an enzyme which efficiently breaks down rice starch into glucose. For clear *sake*, it is important to prevent coloration of *sake* by choosing a strain that does not produce too many spores or pigmentation.

Soy sauce is a liquid seasoning obtained by the hydrolysis of soy proteins into free amino acids. Because the primary role of *koji* mold for soy sauce making is to break down soy proteins, the *koji* mold should have higher protease activities than amylase activities. *A. sojae* is also used for some soy sauce products. This species is known for its high capacity to produce proteases, dark green-colored spores, and short conidiophores. Shorter hyphae are advantageous in that they allow better air permeability due to less intertwining of hyphae and that the *teire* process during *koji* making is easier as *koji* clumps would break up easily.

*A. oryzae* is the primary *koji* mold used in *miso koji*, while *A. sojae* is used in some instances. Strains with moderate protease and amylase activities are selected and maintained. During the fermentation and maturation of *miso*, soy proteins are hydrolyzed to release amino acids that add *umami,* while rice and barley starches are broken down into glucose and other sugars. Therefore, *koji* mold for *miso* making should be able to break down both proteins and starches. The balance between proteinase and amylase activities is the key factor when selecting excellent *koji* mold for *miso*.

#### 4.1.2. Starter *Koji* for *Miso* Making

A variety of *koji* starters are available for *miso* making to meet the requirements of wide-ranging *miso* types that vary in ingredients, ingredient proportions, salt concentrations, and color. In one study, 94 *koji* mold strains were isolated from *koji* starters used for *miso* making, and 92 of them were *A. oryzae*, whereas only two were *A. sojae*, which were isolated from *koji* starters for soybean *miso* [10].

The properties and specifications of *koji* starters include enzyme production ability, growth rate, hyphae length, growing form on steamed rice, color, and aroma. These properties can be divided into those important for handling during *koji* making and those associated with *koji* properties. The properties required for a strain used in soy sauce making are clear: high capability to produce proteases and faster spore formation. For *miso*, the definition is not so clear, but reported properties include

(1) A broader spectrum of protease activities;
(2) About 8% higher amylase activities and 20% higher protease activities compared to brewer’s strains;
(3) Correlations among amylase activities and among protease activities but not between amylases and proteases [11].

These properties are likely the result of artificial selection of strains to meet the diverse needs of *miso*. The strains provided from the starter *koji* manufacturers have undergone selection by *miso* makers. The diverse *miso* qualities, which reflect social trends of the time and consumer preferences, seem to have played key roles in the properties of *koji* mold for *miso* making.

Note that the properties of a starter *koji* strain are not directly linked to the quality of *miso*. The quality of *koji* can vary depending on the handling during the *koji* making process, and the quality of final *miso* products is determined by many factors including soybean processing, *shikomi* proportions, control during maturation, and lactic acid bacteria and yeast activities during maturation.

#### 4.1.3. Safety of *Koji* Mold

The safety of *A. oryzae* in food has been empirically demonstrated by the fact that *A. oryzae* has been used for the fermentation of foods for over a thousand years in Japan. Based on the molecular phylogenetic analysis, *A. oryzae* belongs to the *Aspergillus* section *Flavi*. Because the *Aspergillus* section *Flavi* includes an aflatoxigenic species *Aspergillus flavus*, concerns were raised that *A. oryzae* could also produce aflatoxins. In the 1970s, government research organizations, universities, and manufacturers initiated a collaborative research and found that none of the starter *koji* strains were aflatoxigenic [12].

Later, this result was further confirmed by molecular studies. The aflatoxin biosynthetic gene system of *A. flavus* is made up of a cluster of more than 25 genes. By PCR analysis, 15 of 39 strains of *A. oryzae* were shown to have deletions in five genes within the cluster that is homologous to the aflatoxin biosynthetic gene cluster of *A. flavus*. In the remainder of the strains examined, the genes within the homolog cluster were dysfunctional [13]. In 2005, the whole genome sequencing was completed for *A. oryzae* strain RIB40, and this strain contained the aflatoxin biosynthesis gene homolog cluster. Using the sequences of seven homolog genes (*aflT*, *nor-1*, *aflR*, *norA*, *avnA*, *verb*, and *vbs*) found in this strain, 196 strains of *koji* mold were analyzed by PCR. It was found that they could be classified into three groups: 105 strains having seven of those genes (Group 1), 81 strains having three genes (Group 2), and eight strains having one gene (Group 3), and that half of the strains had deletions in cluster genes [14]. In addition, the expression of *aflR*, which encodes a transcription factor for the entire cluster, was absent in the expressed sequence tag data of *A. oryzae* RIB40, strongly indicating that the aflatoxin biosynthesis gene cluster is unlikely expressed [15]. RT-PCR analysis has also shown that the expression of *aflR* is very low in the 10 strains in Group 1. Furthermore, the expression of *avnA*, *verB*, *omtA*, and *vbs* cluster genes was not detected [14].

Thus, *koji* mold does not produce toxins during culture, and genomic studies have shown deletions in the aflatoxin biosynthesis gene cluster as well as mutations in the transcription factor gene *aflR* and other cluster genes. These findings support that *koji* mold is not toxigenic from both toxicological and genetic perspectives.

### 4.2. Koji Making and Enzyme Production

The characteristics of starter *koji* strains are not the only determinant of the quality of final *miso* products. Process conditions during *koji* making also have a significant influence on enzyme production and coloration of *koji*.

#### 4.2.1. Amount of Starter *Koji*

Unlike industrial microbial culture, *koji* making does not need precultures and starts as soon as starter *koji* is inoculated to steamed rice or barley. Therefore, it is best to inoculate as many spores as possible. For *koji* making for *miso* production, 5 × 10^5^ spores per 1 g of rice is considered optimal. In a laboratory setting, changes in the spore count affect the length of the growth induction phase but not the final enzymatic activities or fungal count in *koji* obtained after culture [16]. In the industrial setting, however, the inoculation amount should be optimized based on production conditions, because the rate of respiratory heat generation affects the temperature control management and enzyme production efficiency.

#### 4.2.2. Blending Different *Koji* Starters

Commercial starter *koji* can be a single strain or a mixture of multiple strains. Multiple-strain *koji* starters are formulated by each manufacturer to achieve the best balance for the growth of each strain based on their experimental data. Some *miso* manufacturers endeavor to add more strains in the hope of incorporating different benefits. Often, however, blending strains with different growth rates would not produce the desired effects, and it could even reduce enzyme production efficiency. Experimental validation is recommended for any blend of *koji* starters before starting commercial application.

#### 4.2.3. Effects of Additives

Powdered starter *koji* is used with additives to ensure uniform distribution, facilitate enzyme production, and enhance ingredients. Calcium carbonate is used for uniform distribution and improved protease production. The addition of sodium phosphate and sodium glutamate, sodium succinate and other compounds has been shown to increase protease production [17].

#### 4.2.4. Conditions for *Koji* Making

The factors that can be artificially controlled during *koji* making are ambient temperature, humidity, ventilation rate, and culture length. The *koji* growth and enzyme production are also affected by these factors, especially by temperature, moisture, and culture length. The optimum temperature and humidity are similar for all *koji* strains and typically 35–38 °C and 95%, respectively. Nonetheless, each strain has its own unique growth rate and enzyme productivity, and the culture environment should be optimized to achieve the desired enzyme profiles.

- Temperature Transition

In *koji* making for rice *miso*, the baseline temperature is 35 °C, and temperature is adjusted accordingly in the last half of the culture when enzymes are produced until the desired enzymatic activities are achieved. For example, the temperature is maintained at 2–5 °C above the baseline for sweet *miso* to increase amylase production. For barley *miso*, it is reduced by 2–5 °C. Temperature adjustment is necessary because the optimum temperature for synthesis is different for each enzyme. Different enzymatic activities are desired for different types of *miso*, and the amount of enzymes to be produced can be adjusted by the temperature during *koji* making. Note that the production of protease enzymes cannot be enhanced at a temperature above 40 °C.

- Ambient Humidity

In *koji* making, the relative humidity around steamed rice is in equilibrium with water activity (Aw) of the moisture contained in steamed rice. Initially, it is 98% and then changes with the Aw of *koji*.

The Aw of *koji* decreases during *koji* making due to (1) water generated through the metabolism of *koji* mold and evaporation of water by metabolic heat, and (2) low molecular weight sugars generated by the breakdown of starch by amylases. At the end of the *koji* making, the lowest limit of Aw (0.90) is reached for *koji* mold growth. Optimal enzyme production is achieved at moisture levels that are slightly lower than those for optimal growth. *Koji* making starts at optimal Aw for growth, undergoes optimal Aw for enzyme production and ends at low Aw that limits the growth [18].

- Duration of *Koji* Making

For each *Koji* enzyme, the time course of synthesis follows a different pattern. By changing the time to finish the *koji* culture, we can adjust the enzymatic contents and activities in the final *koji*. In commercial *koji* plants, however, it is often impossible to change the time as desired due to *shikomi* and other process schedules and employees’ working hours. The duration of *koji* making can be shortened by selecting strains with higher enzyme production efficiency. The enzymatic activities in starter *koji* for *miso* making can vary 2- to 4-fold depending on the strains. High productivity strains can reduce the time required for *koji* making. In addition, increasing the amount of starter *koji* can also shorten the length of the growth induction phase. A combination of these can be used to reduce the entire duration of *koji* making.

### 4.3. Enzymes in Koji

In brewery and fermented food production, the most important role of *koji* is to provide enzymes. In *miso* making, proteins and polysaccharides in soybeans, rice, and barley are hydrolyzed into amino acids and monosaccharides, respectively, which determine the taste, aroma, digestibility, physiological benefits, and other functional qualities of *miso*. Proteases, amylases, and lipases contained in *koji* are responsible for the breakdown of these components in *miso* ingredients. In addition to these enzymes that are already known, extracts of *koji* likely contain almost all types of enzymes. This is why *koji* is called a “gold mine of enzymes”.

The sequence analysis of *A. oryzae* genome was completed in 2005. It was cleared that *A. oryzae* genome contained 13,572 genes and 25% of these genes were identified as unknown genes. Discovery of new and novel enzymes from the *A. oryzae* genome is expected [19].

## 5. *Koji* Making (*Seikiku*) [20]

### 5.1. Rice Koji

#### 5.1.1. Roles of *Koji* Making

Rice *miso* accounts for the majority of *miso* produced in Japan. In this section, we will mainly discuss rice *koji*, which is used in rice *miso*.

*Koji* making has four roles in *miso* making:

- Growth and elaboration of fungal hyphae around and into the ingredients by solid-state culture;
- production of amylases, (neutral) proteases and other enzymes important for *miso* fermentation and maturation (hypha extension into the ingredients (*hazekomi*) enhances this process);
- growth of salt-tolerant yeast and lactic acid bacteria that are essential for maturation and production of precursors of aromatic components in *miso* (the growth of *koji* mold facilitates this process); and
- elimination of ingredient odors.

In addition, the extension of *koji* hyphae creates numerous spaces inside the ingredients and facilitates the hydrolytic actions of enzymes. In addition to amylases and neutral proteases secreted from *koji* mold, other types of enzymes that are retained within the *koji* bodies also facilitate the maturation process. Similarly, intracellular nucleic acid degradation products also contribute to the taste of *miso*.

#### 5.1.2. Growth Conditions of *Koji* Mold in *Koji* Making Process

Three to 5 h after attachment to the solid medium (i.e., steamed rice), starter *koji* spores germinate. The hyphae grow and start to secrete high molecular weight hydrolytic enzymes such as amylase and proteases from their ends. After germination, a lower *koji* temperature is maintained to suppress contamination with *Bacillus subtilis* and other unwanted bacteria. As hyphae grow and extend, respiratory heat generation, oxygen consumption, and carbon dioxide production increase markedly. To encourage healthy growth, it is necessary to increase the ventilation rate to provide adequate oxygen, lower the *koji* temperature, and remove carbon dioxide. As the culture continues, *koji* mold extends its hyphae into rice grains. This is called *hazekomi*. In thoroughly cooked rice, *koji* hyphae can grow deeper into rice grains by hydrolyzing starches. In undercooked rice, in which gelatinization is incomplete, the tip of the hypha cannot dig forward and extends only on the surface of the grain. This results in the insufficient production of enzymes and failure to achieve the enzyme activities needed for *miso* maturation.

#### 5.1.3. Optimal *Koji* Making Conditions for Enzyme Production

The most critical enzymes in *miso* making are amylases, which hydrolyze starches in *miso* ingredients, and proteases, which break down proteins also in *miso* ingredients. For sweeter *miso*, *koji* is used in higher proportions. The ability to increase the production of glucose and oligosaccharides from starches is required to enhance sweetness. For this reason, *koji* is cultured at a relatively higher temperature of 35–38 °C to ensure higher amylase production. For salty *miso*, it is important to have higher protease activities to facilitate hydrolysis of proteins into amino acids and peptides, which constitute *umami* in this type of *miso*. For this purpose, the temperature is maintained at 30 °C or below during *koji* making.

#### 5.1.4. *Koji* Making Methods

*Koji* making is largely divided into manual and mechanical methods. The tray *koji* method is a traditional manual method passed down from generation to generation. In this method, *koji* is cultured in a culture room using wooden trays called *futa* and wooden beds called *toko* for insulation. Because the processing volume is limited, it is not suitable for large-scale *miso* making. However, this method is still used by manufacturers who advertise hand-crafted *miso* making and for experimental *shikomi*. This method consists of loading (*hikikomi*), kneading (*kirikaeshi*), piling (*morikomi*), mixing (*teire*), rearranging (*tsumikae*), and finishing (*de-koji*).

Loading (*hikikomi*): Steamed rice and other ingredients, if used, are cooled down, inoculated with starter *koji* (*tanetsuke*) and laid out on a *toko* bed in a culture room (*hikikomi*). The bed is then covered with cloth to maintain temperature and moisture. The temperature of steamed rice is controlled between 27 and 30 °C.

Kneading (*kirikaeshi*): Spores grow on steamed rice and start to generate heat around 10 h after inoculation, leading to rapid heat generation around the 16th hour. To prevent excessive temperature rise, *koji* is mixed and kneaded by hand. This process is called *kirikaeshi*. It also prevents rice grains from sticking to each other as the mold hyphae continue to grow.

Piling (*morikomi*): After kneading, the batch is transferred to *futa* trays. A thin wooden plate that is slightly raised at its center is laid in each *futa* tray to help heat dissipation.

Mixing (*teire*): After being transferred to *futa* trays, *koji* mold continues to grow. *Koji* is mixed manually before the temperature becomes too high. This *teire* process is usually done twice and is aimed at controlling the temperature and providing oxygen while reducing carbon dioxide concentration.

Rearranging (*tsumikae*): The temperature is not even across the culture room. *Futa* trays are rearranged to even out the temperature history for each tray.

Finishing (*de-koji*): About 40 h after loading, *koji* is taken out of the culture room. The optimal timing of unloading is different for each strain of *koji* mold.

Mechanical/automatic *koji* making.

Mechanical or automatic *koji* making has become popular in recent years to replace labor-intensive manual methods. Various systems are commercially available from different fermentation and brewing machinery manufacturers. These systems are designed to deliver temperature- and humidity-controlled fresh air around and into *koji* to prevent excessive heat accumulation inside the *koji* and to exchange oxygen and carbon dioxide. Depending on the ventilation method used, *koji* making machines are divided into a surface ventilation system and interior ventilation system. The interior ventilation system is further divided into fixed bed, shelf bed, rotating drum, and rotating disc systems depending on how *koji* ingredients are loaded. The apparatus is ventilated before the *koji* temperature reaches 40 °C due to respiratory heat. Humidification of the air may be stopped just before unloading the *koji*.

#### 5.1.5. Quality of *Koji*

The quality criteria desired for *koji* at the time of finishing are as follows:

- Contains enzymatic activities required for the type of *miso* to be manufactured;
- Sufficient depth of *hazekomi* with minimal coloration and brilliant color;
- Aromatic without foul odor from bacterial contamination;
- Fluffy and soft texture;
- Minimal sporulation and coloration with high amylase activities for white or yellow *miso* through shorter culture time; and
- High protease activities through slightly longer culture for red *miso*.

### 5.2. Barley Koji

Barley *koji* tends to generate more respiratory heat than rice *koji*, because barley is richer in protein, inorganic salts, and vitamins. The surface moisture of steamed barley evaporates easily, making barley grains too dry for the optimal growth of *koji* mold. Therefore, in the early phase of *koji* making it is essential to maintain the humidity close to the saturation point and deliver highly humid air at a temperature as close as possible to the *koji* temperature. At the same time, caution needs to be taken because sticky barley with too much moisture facilitates the growth of unwanted bacteria. For light-colored barley *miso* containing a high proportion of *koji*, the *koji* temperature is controlled to reach 36–38 °C at 10 to 18 h after loading to obtain higher amylase activities. It is then decreased to 30–32 °C. For red barley *miso*, on the other hand, high temperature must be avoided to enhance both protease and amylase activities. It is usually controlled at a slightly low temperature around 29–31 °C. Occasionally, finished *koji* is mixed with salt to prevent continued heat generation during storage. Finished *koji* is mixed with salt (about 1/3 of salt used in the initial ingredients) to suppress respiratory heat generation. Salted *koji* must be used within 2 days, because enzymatic activities will decrease.

### 5.3. Soybean Koji

To make rice and barley *miso*, rice and barley are steamed, inoculated with starter *koji*, and mixed with salt and soybeans. To make soybean *miso*, soybeans are steamed, cooled down to 40 °C, and made into *miso* balls using a specialized machine called *tamanigiri-ki* and then used for *koji* making. Different sizes and shapes of *miso* balls are used at each manufacturer. The reason for this extra step is to facilitate the growth of facultative anaerobic lactic acid bacteria inside the balls to lower pH and prevent the growth of *B. subtilis*, which has a negative effect on the growth of *koji* mold.

Although some manufacturers prefer 45 mm or larger *miso* balls, most common *miso* balls are 19–24 mm in diameter. Larger *miso* balls have more space to grow facultative anaerobic lactic acid bacteria, resulting in significantly higher amounts of lactic acid, which tend to delay the maturation of *miso*. To prevent *miso* balls sticking to each other, *miso* balls are usually coated with roasted and powdered barley called *kosen* or *hattaiko* for better aeration. During *koji* making, a lower temperature is maintained in the early phase to suppress the growth of *B. subtilis* and then increased to dissipate the moisture from *miso* balls to induce the growth of *koji* mold into the balls.

## 6. *Koji* Enzymes Involved in *Miso* Making

The degradation of starches and proteins in soybeans, rice, barley, and naked barley is the key process in *miso* making. This is where *koji* enzymes work. *A. oryzae* is the main *koji* mold used in *miso* making [21]. *A. oryzae* produces amylases (e.g., Taka-amylase and glucoamylase) and proteinases in large quantities [22]. To make *miso*, *koji*, salt water, and steam-boiled grains are mixed. The high salt content and anaerobic conditions prohibit the growth of *koji* mold, and only the enzymes produced during *koji* making can act on the ingredients.

Starches in the *miso* ingredients are broken down into glucose by Taka-amylase and glucoamylase. These glucose molecules allow the growth of salt-tolerant yeasts *Z. rouxii* and *Candida versatilis*, and alcohols produced by these yeasts enhance the flavor of *miso* [23]. On the other hand, proteins are made up of 20 types of amino acids, which have side chains with different properties and size. No single protease can hydrolyze all of the peptide bonds formed between these different types of amino acids. Thus, more diverse types of proteases are involved in *miso* making compared to amylases, and genomic analysis has identified about 130 different proteases in *A. oryzae* [24]. Because protein breakdown is especially important in *miso* making, this section focuses primarily on proteinases in *koji* mold.

Because the pH remains around 5 to 6 during *miso* maturation, proteases that work in a weakly acidic environment are the key. Acid proteases likely play a major part, while neutral and alkaline proteases may also be involved [25]. For exopeptidases, acid carboxypeptidase and a trace amount of leucine aminopeptidase seem to have an important role.

### 6.1. Acidic Endopeptidases

Genomic analysis of *koji* mold identified two acidic proteases, aspartic endopeptidase and glutamic peptidase [24]. As a mammalian protease belonging to the pepsin family, aspartic endopeptidase has two aspartate residues in its active site and is optimally active at around pH 3. *Koji* mold has 11 aspartic endopeptidase genes, and sequence homology analysis of encoded amino acids suggests that five are extracellular, three are vacuolar, and three are glycosylphosphatidylinositol-anchored. In the making of clear rice wine and *shochu* (Japanese distilled spirits), PepO (PepA), the major aspartic endopeptidase in *koji* mold, has been shown to break down rice proteins to release amino acids and peptides [26,27,28]. PepO appears to preferentially hydrolyze peptide bonds where hydrophobic and aromatic amino acid repeats are present. It is thought that as the acidity and alcohol concentration increase, substrate proteins are denatured, and hydrophobic side chains become exposed and accessible to the enzyme [29].

In a recent animal study, *A. oryzae*-derived PepO has shown a promising role as a prebiotic, and dietary supplementation of a very small amount of PepO significantly increased beneficial *Bifidobacterium* [30]. It has been reported that *miso* retains acid protease activities for a long period [31,32] indicating that the consumption of *miso* may have a similar prebiotic effect.

Aorsin is a member of the sedolisin family and has an optimum pH of around 4 [33]. *Koji* mold carries two aorsin genes, and enzymological properties have been reported for aorsin A. Aorsin A primarily cleaves the peptide bond C-terminal to the arginine residue. The second aorsin (aorsin B) is specific to aspartic and phenylalanine residues [29].

### 6.2. Neutral Endopeptidases

*A. oryzae* contains genes that encode neutral endopeptidases, fungalysin, and deuterolysin. Fungalysin is a thermolysin-type metalloendopeptidase with Zn^2+^ in the active center shared by filamentous fungi [34]. *Koji* mold has two enzymes, neutral protease I (NpI), and neutral protease III (NpIII). Whereas NpIII is yet to be characterized, NpI is most active at around pH 7 and specifically cleaves a peptide bond at the C terminus of hydrophobic and bulky amino acid repeats [29].

Deuterolysin is also a metalloendopeptidase with Zn^2+^ in its active center, but Zn^2+^ is positioned at a different amino acid [35]. Its molecular weight is about 19 kDa, much smaller than fungalysin (~45 kDa). The optimum pH is 7 to 8. Two deuterolysins, DeuA and DeuB, are found in *koji* mold [36]. DeuA, also called NpII, is highly thermostable and still active after 10 min of heat treatment at 100 °C [37]. In contrast, DeuB is not heat-tolerant and almost completely inactivated after 10 min at 80 °C. Because DeuA is mainly produced in solid-state culture, DeuA is considered the major deuterolysin in *miso* making. These enzymes efficiently break down basic proteins such as salmine, clupeine, and histone, but their efficiency is very low for casein and hemoglobin. This is why these enzymes are often overlooked in casein-based enzyme activity studies.

### 6.3. Alkaline Endopeptidases

Oryzin is an extracellular alkaline protease produced by *koji* mold [38,39]. It is optimally active at a pH of 10 to 10.5 and hydrolyzes a peptide bond at the C terminus of hydrophobic and aromatic amino acids with bulky side chains, such as leucine, tyrosine and phenylalanine.

### 6.4. Exopeptidases

During *miso* maturation, proteins in *miso* ingredients are broken down by the endopeptidases discussed above, and the resulting peptides are further broken down into amino acids and oligopeptides by exopeptidases.

The most important exopeptidases in *miso* making are said to be serine-type carboxypeptidases. These enzymes cleave the C-terminal amino acid one by one, and the released free amino acids are thought to play an important role in *umami* and flavor formation in *miso*. Ten extracellular secreted enzymes belonging to the MEROPS (the peptidase database; https://www.ebi.ac.uk/merops/, accessed on 7 May 2021) S10 family have been identified in *koji* mold [40]. Of these, carboxypeptidase I from *A. oryzae* strain TK3 [41], OcpO specifically found in a liquid culture of *A. oryzae* strain IAM2640 [42], carboxypeptidases O1 and O2 found from solid-state cultures [43], and their orthologs in the strain RIB40, CpI, OcpO, OcpA, and OcpB [44,45], have been enzymologically characterized. The optimum pH is below 4 for all of these enzymes. Their specificity is somewhat different, and the optimum substrates are Z-Tyr-Leu and Z-Phe-Leu for OcpO, Z-Phe-Leu for OcpA and Z-Phe-Leu and Z-Leu-Tyr for OcpB.

Aminopeptidases also likely have a role in releasing amino acids from peptides. Aminopeptidases cleave the N-terminal amino acid one by one at neutral pH. It is estimated that 23 different aminopeptidases are present in *koji* mold, and four of them are secreted into the extracellular space. All of these enzymes belong to the metallopeptidase family. The two leucine aminopeptidases, LapA and LapB, share 56% identity in their amino acid sequences [46,47]. LapA has an optimum pH of around 8.5 and cleaves hydrophobic and basic amino acids such as leucine, phenylalanine, methionine, lysine, and arginine but not acidic amino acids such as aspartic acid. LapB has a wider substrate specificity with strong affinity for leucine, lysine, alanine, and glutamic acid and also cleaves valine, proline, and isoleucine. Its optimum pH is alkaline, between 9.5 and 10. The other two enzymes have been partially isolated and likely have a similar substrate specificity to LapA according to Kusumoto et al. [29].

In addition to aminopeptidases, *koji* mold also contains dipeptidyl peptidases and tripeptidyl peptidases that catalyze the cleavage of the terminal peptide bond. Although both peptidases have serine in the active site, dipeptidyl peptidases are serine endopeptidases, while tripeptidyl peptidases belong to the sedolisin family.

Dipeptidyl peptidases release a dipeptide from the N terminus one by one, and three extracellular enzymes, DppB, DppE, and DppF, are found from *A. oryzae* [48]. The optimum pH is around 7 for all of these enzymes. DppB acts on substrates in which the second N-terminal amino acid is proline, and it is a homolog of the mammalian dipeptidyl peptidase IV first discovered by Tachi et al. [49]. Compared to DppIV previously found in *A. fumigatus*, DppB shows a stricter substrate specificity and unlikely acts on substrates if the second N-terminal amino acid is not proline. It works more preferentially when the N-terminal amino acid is arginine, alanine, and glycine in this order, indicating that the size and polarity of the side chain may influence the specificity. DppE and DppF, which correspond to DppV of *A. fumigatus*, work more actively when the second N-terminal amino acid is alanine or phenylalanine. These two enzymes may work synergistically, as their specificities are different.

### 6.5. Pro-Xaa Peptidases

Recently, four peptidases (AoS28A, AoS28B, AoS28C, and AoS28D) belonging to the MEROPS S28 family have been newly identified from *A. oryzae* [50]. This family contains human lysosomal Pro-Xaa endopeptidase. AoS28A and AoS28B hydrolyze a peptide bond at the C terminus that contains proline and have an optimum pH of 4 and 4.5, respectively. Their specificities are different and determined by amino acids flanking the proline residue, indicating that they work synergistically. On the other hand, AoS28D is a Pro-Xaa carboxypeptidase that cleaves the C-terminal amino acid when the second C-terminal amino acid is proline [51]. The serine-type carboxypeptidases discussed above do not have these catalytic activities, but AoS28D can release Xaa from -Pro-Xaa at pH 3.8 and 7.0 and also Xaa-amide from -Pro-Xaa-amide. It can release C-terminal amino acids including phenylalanine, isoleucine, threonine, glutamine, glutamic acid, arginine, and lysine but cannot release proline from -Pro-Pro. Thus, AoS28 enzymes are capable of hydrolysis of the proline-containing C-terminal peptides, which are usually difficult to hydrolyze. It is likely that AoS28A and AoS28B evolved as endopeptidases, while AoS28D evolved as an exopeptidase.

### 6.6. Glutaminase

Glutamic acid is the *umami* component in soy sauce and *miso*. Glutamic acid is one of the amino acids released by protein hydrolysis [52]. It has been shown that *Koji* mold not only releases glutamic acid from proteins, but also converts glutamine to glutamic acid enhancing *umami* during soy sauce making. Glutaminase is the enzyme responsible for the conversion of glutamine to glutamic acid. It catalyzes the hydrolysis of glutamine into glutamic acid and ammonia. Twelve glutaminase genes are present in *A. oryzae* [53]. Because these include orthologs of GahA and GahB found in *A. sojae*, which convert glutamine to glutamic acid at the C terminus, *A. oryzae* likely increases glutamic acid through a similar mechanism [54].

## 7. *Z. rouxii* and *T. halophilus* in *Miso* Making

The main microbes working during the fermentation and maturation process of *miso* are yeast and lactic acid bacteria, which are usually added at the time of *shikomi*. *Z. rouxii* plays a main part, while *C. versatilis* and *C. etchellsii* are also working in late maturation. *Z. rouxii* is a heterothallic, spore-forming yeast. *C. versatilis* and *C. etchellsii* are non-spore-forming yeasts that grow in the late maturation phase and produce aromatic components. In general, *miso* yeast is the term referring to *Z. rouxii.*

Under aerobic conditions, *Z. rouxii* breaks down glucose into carbon dioxide gas and water and grows rapidly using glucose as energy. Under anaerobic conditions, it shifts to the fermentation of glucose and produces alcohol and carbon dioxide gas. Glycerol, succinic acid and acetaldehyde are also produced as by-products. Glycerol and succinic acid add depth to *miso* taste, while acetaldehyde masks the ingredient odor. Alcohol reacts with organic acids to form various esters. *Z. rouxii* also produces higher alcohols such as isoamyl alcohol by decarboxylation of amino acids.

*Z. rouxii* is tolerant to up to 4M of salt, and as the salt concentration increases, the optimum growth pH narrows down to 5.0.

The main lactic acid bacterium used in *miso* making is *T. halophilus*, which is a Gram-positive, non-spore-forming, homofermentative tetrad cocci. It was once called *Pediococcus halophilus* as proposed by R. H. Mees in 1934 until its scientific name was changed to *Tetragenococcus halophilus* in the 1990s [55]. It is a halophilic bacterium that grows most efficiently at 5–10% salt and can survive with a higher salt concentration up to 24%. The optimum growth temperature is 25–30 °C, and the optimum growth pH is between 5.5 and 9.0. It is sensitive to acidic conditions. *T. halophilus* is characterized by wide-ranging properties. For example, the amount of lactic acid production is influenced by sugar and oxygen levels [56], and different strains show different sugar fermentation efficiency and amino acid hydrolysis [57,58].

## 8. Changes in *Miso* Components during Fermentation and Maturation

### 8.1. Macronutrients

Macronutrient compositions of finished commercial *miso* products are shown in Table 1. The water content may vary from 40 to 50%, but it is generally between 44 and 46%. The salt content varies widely from 5 to 13%, but typically it is in the 11 to 13% range. The water and salt are the important factors that change the components in *miso* and play various roles such as inhibition of unwanted microbial growth, promotion of growth and metabolism of fermentation microbes and control of enzymatic actions.

The pH of *miso* is around 6 at the start of fermentation/maturation but decreases to around 5 during maturation. This is the reason pH is used as a process parameter that indicates the degree of maturation. The Aw also decreases during maturation, and it is usually 0.80 ± 0.05 in matured *miso*.

The protein content of rice *miso* is generally between 12 and 13%. Soon after the ingredients are mixed, proteins are degraded rapidly, releasing a large quantity of free amino acids during early maturation. Matured *miso* is rich in free glutamic acid, arginine, lysine, and leucine [60].

Carbohydrates in *miso* are mostly starches from rice and also include polysaccharides such as arabinogalactan from soybeans.

Lipids in *miso* are mainly derived from soybeans and are typically around 6% in rice *miso*. During maturation, lipase from *koji* mold hydrolyzes some lipids into fatty acids and glycerol. A part of the fatty acids reacts with ethanol, which is generated by yeast fermentation, to form ethyl esters, the important constituents of *miso* aroma.

### 8.2. Organic Acids

Lactic acid, acetic acid, citric acid, and succinic acid make up a large part of the organic acids in *miso*. Lactic acid and acetic acid are produced by lactic acid bacteria, while a small quantity of succinic acid is generated by lactic acid bacteria and yeast. Citric acid is derived from soybeans and remains in *miso*. Lactic acid bacteria utilize citric acid to generate more lactic acid and acetic acid. This is how lactic acid, acetic acid and succinic acid continue to increase during the maturation of rice *miso*.

### 8.3. Color and Aromatic Compounds

The Maillard reaction (amino-carbonyl reaction) plays a large part in component changes during *miso* maturation. First described by L. C. Maillard in 1912, the Maillard reaction is a non-enzymatic, browning reaction between amino compounds and carbonyl compounds resulting in the formation of high-molecular weight polymers, melanoidins. The Maillard reaction not only adds color, but also generates a range of aromatic compounds.

The characteristic rich and mellow aroma of *miso* is known for its susceptibility to change during fermentation/maturation and heat treatment. Over 200 aromatic compounds have been identified from rice *miso* [61]. 4-Hydroxy-2 (or 5)-ethyl-5 (or 2)-methyl-3 (2H)-furanone (HEMF) that produces a sweet caramel-like aroma with a very narrow threshold value has been extensively studied. HEMF is associated with the sensory analysis results of rice *miso*, and it is identified as one of the critical compounds responsible for the appealing aroma of *miso* [62]. In rice *miso*, HEMF is not detected immediately after the mixing of *miso* ingredients but starts to increase with maturation and then decreases during the late maturation phase [63]. 4-Ethylguaiacol is an important aroma component that allows us to distinguish *miso* types containing different *koji*, and HEMF and methionol are important aromatic components produced by yeast. The combination and concentrations of these three components seem to determine the aromatic property of *miso* [64]. More recently, Kumazawa et al. [65] identified 16 new aromatic components in rice *miso* and found that trace amounts of low-threshold components other than HEMF also contribute to *miso* aroma. Further understanding of aroma components in *miso* awaits future research.

## 9. Nutritional Function of *Miso*

The health benefits of *miso* have been passed down orally through hundreds of years by way of proverbs and folklore. Due to its high salt content, however, specific health benefits of *miso* have been discussed with caution in modern medicine and public health. In fact, restricted *miso* consumption is sometimes recommended in Japan to reduce salt intake. While *miso* has recently become a popular research topic as a functional food [66], studies are mostly conducted in cell cultures and laboratory animals and rarely involve human participants. Two published studies of rice *miso*, however, found that long-term standard consumption of *miso* soup did not affect blood pressure in human subjects [67,68]. While growing evidence suggests no association between *miso* soup consumption and high blood pressure [69], multipronged studies are necessary to understand the exact mechanisms.

Cohort studies are another approach to examine the benefit of *miso* as a part of regular Japanese diet. In a recent cohort study [70], Katagiri et al. followed 92,915 men and women aged between 45 and 74 years, who responded to dietary habit surveys conducted between 1995 and 1998, to prospectively evaluated the relationship between the risk for mortality and consumption of soybean products and fermented soybean products up to 2012. The study showed that while no association was found between the risk of death and consumption of all types of soybean products combined, the risk of death decreased with increasing consumption of fermented soybean products in both men and women.

Dietary sodium intake is much higher in the Japanese population than in Western countries, and the majority of it comes from seasoning. However, a study by Okada et al. [71] has shown that the portion size of soy sauce or *miso* is not associated with hypertension. These results were based on 25,738 Japanese men and women aged 20 years or older who participated in the national health and nutrition surveys between 2012 and 2016. The study explains that people who consumed soy sauce or *miso* in larger portion sizes more likely consumed larger quantities of vegetables, fruits, and fish, resulting in a higher intake of potassium, which has been shown to decrease blood pressure.

One of the nutritional advantages of *miso* is that soy proteins and starches are predigested and easily absorbed as amino acids and saccharides. In particular, because *miso* is eaten as a soup or as *aemono* (chopped fish, shellfish, or vegetables, dressed with sauce such as *miso* or vinegar), it is an excellent companion to a balanced diet consisting of vegetables, seafood and meat. The health benefits of this type of diet are beyond calculation.

## 10. Conclusions

Globally, soybeans are the dominant oilseed crop, while in Japan they are considered as an important food source. Especially, *miso* is an essential part of the Japanese diet as it contains a wide variety of nutritious fermentation products derived from soybeans and grains as a result of the activities of *koji* enzymes and beneficial microbes. It is truly a gift from the Japanese tradition.

We highly anticipate that the increasing popularity of *miso* in the world will play a role in facilitating further globalization of Japanese culinary culture.

## Figures and Tables

**Table 1:** Concentration of nutrients in *miso* (per 100 g) [59].

Nutrients	Rice *Miso*, Sweet	Rice *Miso*, Light Yellow	Rice *Miso*, Red	Barley *Miso*	Soybean *Miso*
Water (g)	42.6	45.4	45.7	44.0	44.9
Protein (g)	9.7	12.5	13.1	9.7	17.2
Lipid (g)	3.0	6.0	5.5	4.3	10.5
Carbohydrate (g)	37.9	21.9	21.1	30.0	14.5
Dietary fiber, total (g)	5.6	4.9	4.1	6.3	6.5
Salt equivalents (g)	6.1	12.4	13.0	10.7	10.9
